# Efficacy and safety of the use of omalizumab in a patient with difficult-to-control severe asthma and antiphospholipid antibody syndrome

**DOI:** 10.1186/1939-4551-8-S1-A219

**Published:** 2015-04-08

**Authors:** Rosangela Villela, Janaina M Melo, Thais Nociti, Ana Carla S Araujo, L Karla Arruda

**Affiliations:** 1 School of Medicine of Ribeirao Preto - University of Sao Paulo, Brazil; 2 Brazilian Association of Allergy and Immunology, Brazil

## Background

Autoimmune conditions may be associated with allergic diseases. There are few studies on the safety of omalizumab use in patients with asthma who also present autoimmune disease.

## Methods

Case report of a patient with severe asthma associated with antiphospholipid antibody syndrome (APS) treated with omalizumab.

## Results

Female, 36-year-old patient, with uncontrolled asthma, dependent on oral corticosteroid. Patient had a history of cough, wheezing and dyspnea since childhood with worsening in the past few years, with dyspnea on minimal activities and use of rescue albuterol 3-10 times a day. She had a history of intensive care unit admission and intubation for asthma on two occasions. Associated conditions were: APS (thrombosis of the leg, miscarriage, stillbirth and positive lupus anti-coagulant, use of aspirin, depression, GERD, and allergic rhinitis. She was on Formoterol/Budesonide 12/400mcg plus inhaled Beclomethasone 800mcg twice daily; Montelukast 10mg a day; Prednisone 5mg daily; Fluoxetine 40mg daily; nasal Budesonide 50mcg twice a day; Omeprazole 20mg once a day.

Her initial spirometry showed an FEV1 of 47% predicted, with response to bronchodilator (21% and 300mL reversibility). This pattern remained throughout her follow up in our Clinic. Her total IgE was 120kU/L, and she presented positive skin prick tests to *Dermatophagoides pteronyssinus*, *D. farinae* and *Trichophyton* and negative to *Aspergillus*. Chest tomography and bronchoscopy were unremarkable.

Despite correct use of medications, the patient had frequent exacerbations and need for increasing doses of oral prednisone up to 80mg daily, with weight gain of 18kg in 3 years. Omalizumab was started, and within two weeks the patient showed marked improvement of symptoms, making it possible to withdraw prednisone. There was reduction in use of rescue albuterol, improvement in quality of life and weight loss. After 9 months, treatment with omalizumab was discontinued due to supply problems, with worsening of asthma and return of oral corticosteroids. Provision of omalizumab was restored after 6 months, and the patient again had improvement of symptoms and discontinuation of oral corticosteroids. Currently, she uses rescue bronchodilator 2 times a week, lost 20kg and practices physical activity.

## Conclusions

Omalizumab was very effective in controlling symptoms in a patient with severe asthma and APS. No adverse effects related to medication use were observed.

## Consent

Written informed consent was obtained from the patient for publication of this abstract and any accompanying images. A copy of the written consent is available for review by the Editor of this journal.

